# Competition between Intramolecular and Intermolecular Interactions in an Amyloid-Forming Protein

**DOI:** 10.1016/j.jmb.2009.04.042

**Published:** 2009-06-19

**Authors:** Katy E. Routledge, Gian Gaetano Tartaglia, Geoffrey W. Platt, Michele Vendruscolo, Sheena E. Radford

**Affiliations:** 1 Astbury Centre for Structural Molecular Biology, Institute of Molecular and Cellular Biology, University of Leeds, Leeds LS2 9JT, UK; 2 Department of Chemistry, University of Cambridge, Lensfield Road, Cambridge CB2 1EW, UK

**Keywords:** β_2_m, β_2_-microglobulin, ThT, thioflavin T, amyloid, aggregation, NMR relaxation, prediction, unfolded state

## Abstract

Despite much progress in understanding the folding and the aggregation processes of proteins, the rules defining their interplay have yet to be fully defined. This problem is of particular importance since many diseases are initiated by protein unfolding and hence the propensity to aggregate competes with intramolecular collapse and other folding events. Here, we describe the roles of intramolecular and intermolecular interactions in defining the length of the lag time and the apparent rate of elongation of the 100-residue protein human β_2_-microglobulin at pH 2.5, commencing from an acid-denatured state that lacks persistent structure but contains significant non-random hydrophobic interactions. Using a combination of site-directed mutagenesis, quantitative kinetic analysis and computational methods, we show that only a single region of about 10 residues in length, determines the rate of fibril formation, despite the fact that other regions exhibit a significant intrinsic propensity for aggregation. We rationalise these results by analysing the effect of incorporating the conformational properties of acid-unfolded β_2_-microglobulin and its variants at pH 2.5 as measured by NMR spectroscopy into the Zyggregator aggregation prediction algorithm. These results demonstrate that residual structure in the precursor state modulates the intrinsic propensity of the polypeptide chain to aggregate and that the algorithm developed here allows the key regions for aggregation to be more clearly identified and the rates of their self-association to be predicted. Given the common propensity of unfolded chains to form non-random intramolecular interactions as monomers and to self-assemble subsequently into amyloid fibrils, the approach developed should find widespread utility for the prediction of regions important in amyloid formation and their rates of self-assembly.

## Introduction

A wide range of highly debilitating diseases are associated with the failure of proteins to maintain their native structures and remain soluble—an abnormal behaviour that results in their aggregation into fibrillar assemblies.^1^ These amyloid fibrils exhibit a common cross-β structure^2,3^ despite the lack of sequence and structural similarities between their precursor proteins.^1^ It has been suggested that the ability to form the characteristic cross-β structure of amyloid is an inherent latent property of polypeptide chains, as many proteins not known to be involved in disease can produce amyloid-like fibrils *in vitro* under appropriate conditions.^3^ Therefore, studying the determinants of fibril formation using model systems can provide important insights into how proteins assemble into the amyloid fold, with applications for understanding both advantageous^4,5^ and deleterious^1,6^ consequences of this protein self-assembly process.

**Fig. 1:**
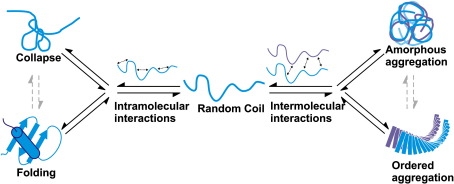
Schematic illustration of the competition between intramolecular and intermolecular interactions in protein folding and aggregation. When intramolecular interactions prevail, proteins form partially or fully folded states (left pathway). By contrast, when intermolecular interactions dominate, protein aggregation results (right pathway).

Whilst insights into the atomic details of cross-β spines of peptides assembled into amyloid-like structures have been obtained,^7–10^ the molecular mechanisms of self-assembly into amyloid fibrils remain poorly understood. One aspect that has been clarified is that the same fundamental forces that lead to folding, which include hydrogen bonding as well as hydrophobic and electrostatic interactions, are also responsible for aggregation. Indeed, native states play a protective role by sequestering in their interior the most aggregation-prone regions of amino acid sequences.^11^ As a consequence, fibril formation may be inhibited by increasing the stability of the native state through mutational analysis or the addition of small molecules.^12,13^ Conversely, therefore, the native state must be destabilised to allow intermolecular interactions that are favourable for aggregation to occur.^11,14–18^ Hence, even in the absence of folding to a native conformation, intramolecular interactions including fluctuating side chain–side chain interactions commonly associated with non-native collapsed states could play a role in modulating the aggregation propensity of a polypeptide chain (Fig. 1).

To investigate this hypothesis, we carried out a systematic analysis of the aggregation kinetics at low pH of β_2_-microglobulin (β_2_m) and 30 mutational variants. Under these conditions, β_2_m forms fibrils with > 90% yield and reproducible kinetics,^19,20^ commencing from a precursor that is unfolded but contains significant non-random structure involving two hydrophobic clusters stabilised by the native disulfide bond.^21^ The results show that only a single region involving residues ∼ 60–70 in the full-length, 100-residue protein plays a major role in determining the kinetics of fibril formation, by contrast with some, but not all, predictions inferred from peptide studies^22–24^ and numerical algorithms.^25–27^ By taking into account the presence of non-random residual structure in the sequence of β_2_m at low pH provided by *R*_2_ measurements^21^ and its dependence on the protein sequence, we have developed an enhanced prediction method based on the physicochemical properties of the amino acid sequence^11^ modulated by its propensity to form non-random intramolecular contacts (Fig. 1). We show that this approach extends the model that we proposed previously for folded states^11^ in providing an accurate description of the competition between intramolecular interactions and aggregation under non-native conditions. Application to the case of β_2_m at low pH demonstrates that this method accurately predicts the regions essential for amyloid formation and in controlling its rate. Given the generic propensity of unfolded chains to collapse and the prevalence of these structures in amyloid precursors of medical importance,^28^ the method developed should find widespread utility for the prediction of β-aggregation in other systems.

## Results

### Design of β_2_m variants

**Fig. 2:**
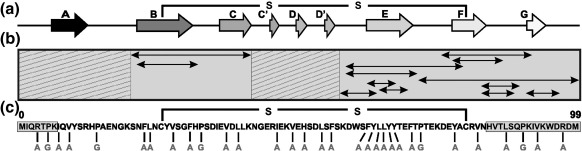
Sequence and fibrillogenic properties of β_2_m and its peptide fragments. (a) Secondary-structure elements of native human β_2_m. The native β-strands (arrows) and the position of the disulfide bond are shown. (b) Regions (double-headed arrows) of β_2_m that have been shown to form fibrils *in vitro* when created as peptide fragments.^7,22–24,29–31^ Note that different conditions were utilised in different studies. Hashed regions (residues 1–19 and 41–59) have not been shown to form fibrils under any conditions tested thus far. (c) Primary sequence of recombinant wild-type β_2_m showing the position and type of amino acid substitutions introduced in this study. The residues at the N- and C-termini truncated to form ΔN6 and ΔC83 are highlighted in grey. The disulfide bond was maintained in all analyses.

Under native conditions, β_2_m forms a disulfide-bonded immunoglobulin fold (Fig. 2a).^32^ By contrast, at pH 2.5, β_2_m is denatured, forming a non-random collapsed state that lacks ordered secondary structure but contains two non-native hydrophobic clusters (residues 29–51 and 58–79) that are mutually interacting and stabilised by the native disulfide bond.^21^ Under these conditions, β_2_m rapidly and spontaneously assembles into fibrils with the morphological, tinctorial, structural and kinetic characteristics of amyloid.^20,33^ Data collected under a variety of different experimental conditions suggest that β_2_m is highly amyloidogenic, with more than 60% of the sequence observed to form amyloid-like fibrils when created as peptide fragments^7,22–24,29,30^ (Fig. 2b). The use of single point substitutions and chimeric proteins also suggested that numerous individual residues and/or regions of the protein are involved in fibril formation (see Ref. 34 for a collated list of the mutations of β_2_m made to date). These results highlight the potential importance of much of the sequence of β_2_m for fibril formation. How this is modulated in the context of the intact protein, however, remains unknown. To address this question, a systematic mutagenesis study is required to resolve residue-specific details of the amyloidogenic determinants of β_2_m in the context of the full-length, oxidised protein under a single, identical set of experimental conditions.

In an earlier study, we investigated the influence of just one region of β_2_m (residues 60–70, previously suggested as a highly amyloidogenic sequence^22,26,35^) in determining the rate of aggregation of the full-length protein.^19^ Building on these results, we describe here a systematic analysis of the aggregation properties of wild-type β_2_m and 30 variants that span its 100-residue sequence, including residues involved in all of the native β-strands or their interconnecting loops (Fig. 2c and Supplementary Table 1). In the majority of the variants created, hydrophobic residues were substituted with alanine. Additionally, the five prolines were individually substituted with glycine. Finally, two truncation variants were created: ΔN6, a species found in *ex vivo* deposits and known to have increased amyloid potential *in vitro*,^36^ and a novel variant, ΔC83, in which residues 84–99 were removed. The C-terminal 28 residues of β_2_m were shown to be highly amyloidogenic when created as a peptide fragment^30^ and to contain a region (residues 83–89) that determines the vastly different amyloid potentials of murine and human β_2_m.^31^

### Role of individual residues in the rates of fibril nucleation and elongation

**Fig. 3:**
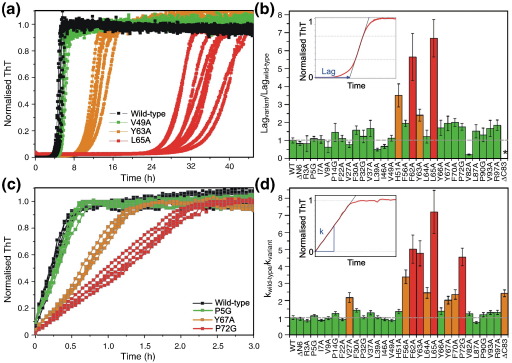
Comparison of the fibrillation kinetics of different β_2_m variants compared with the wild-type protein. Non-seeded (a) and seeded (c) fibril growth kinetics of wild-type β_2_m and representative variants measured using ThT fluorescence. Assays were carried out as previously described (Materials and Methods).^19^ (b) Normalised lag times (Lag_variant_/Lag_wild type_) determined in unseeded growth experiments. The variants are grouped according to changes in lag time compared with wild-type β_2_m; < 2-fold change, green; 2- to 4-fold change, orange; > 4-fold change, red. ^⁎^Under these conditions, fibrils were not formed within the time frame of these experiments (72 h). Inset: illustration of the method used to calculate the lag time. (d) Relative apparent elongation rate (*k*_wild type_/*k*_variant_) determined from seeded growth experiments (see Materials and Methods). The data are coloured as in (b). Inset: illustration of the method used to calculate the apparent elongation rate (*k*). Error bars are ± 1 SD.

**Fig. 4:**
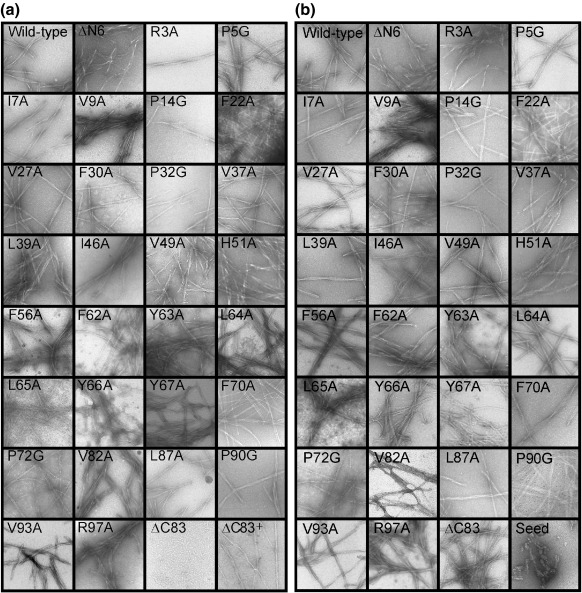
Negatively stained electron micrographs of the end products of fibril formation for wild-type β_2_m and 30 variants. (a) End products of non-seeded fibril formation (72 h). ΔC83 fibrils were generated under different conditions from the other variants (ΔC83^+^) (Materials and Methods). (b) End products of seeded growth of each variant from wild-type seeds. Each square measures 0.5 μm × 0.5 μm.

To assess the effect of each mutation on the kinetics of fibril formation, we monitored the length of the lag phase and the apparent rate of elongation using thioflavin T (ThT) fluorescence as a probe of fibrillogenesis, either in the presence or in the absence of fibril seeds (Fig. 3). Conditions that enable reliable measurement of the rate of fibril formation over multiple samples recorded simultaneously were used (Materials and Methods).^19^ Using this procedure, we determined the mean and variance of the lag time and apparent elongation rate of each variant (Fig. 3). The kinetics of fibril formation of wild-type β_2_m were recorded concurrently with each variant and used to normalise the data obtained. With the exception of the variant ΔC83, all proteins formed fibrils in unseeded reactions within 72 h, each resulting in fibrils that are morphologically indistinguishable by negative-stain electron microscopy from those formed from the wild-type protein (Fig. 4a). Under different incubation conditions (Materials and Methods), ΔC83 also formed long, straight fibrils (Fig. 4a). The lag time of ΔC83 aggregation, therefore, was not compared directly with the other variants. The data nonetheless suggest that the presence of the C-terminal region is important, but not critical, in the initial stages of fibril formation. For the other mutant proteins, the effect of sequence variation on the lag time is dependent on the site of substitution and the type of modification introduced. In particular, five of the variants tested (H51A, F62A, Y63A, L65A and V82A) showed marked differences (> 2- to 7-fold changes) in the average lag time compared with that of wild-type β_2_m, whilst for the other 24 variants, the lag time was relatively unperturbed (resulting in ≤ 2-fold change in the lag time upon mutation) (Fig. 3b and Supplementary Table 1). All the P to G variants showed little difference in lag time compared with wild-type β_2_m (< 2-fold change) despite the fact that substitution of Pro with Gly probes the effects of altering both the main chain and side chain in determining aggregation (Fig. 3b and Supplementary Table 1). Interestingly, L65A has the greatest effect on the lag time, whilst identical amino acid substitutions elsewhere in the sequence result in small (< 2-fold) changes (e.g., compare the data for L65A with L87A) (Fig. 3b and Supplementary Table 1). The only variant to have a significantly shortened lag time compared with wild-type β_2_m was V82A. Why no other residue showed such behaviour is not clear: further substitutions would be needed to explore this further. Together, the data show that for this 100-residue protein, only residues 51, 62, 63 and 65 appear to be critically involved in determining the lag time of fibril formation, with other residues, including those in the C-terminal half of the polypeptide chain, playing a supporting role. This observation contrasts with the behaviour of peptide fragments, which suggested that three regions (residues 20–40, 60–70 and ∼ 80–100) are important for fibril formation^7,22,29,30^ (Fig. 2b). Furthermore, the region of the protein containing His51 has not been observed to be prone to fibril formation as part of an isolated fragment, yet this residue appears to play a significant role in determining the length of the lag phase in the conditions tested. These results indicate that there are fundamental differences in aggregation potential of particular residues or certain sequences when isolated as peptides compared with their behaviour when included within the context of the full-length protein.

Seeds formed by fragmenting fibrils prepared from the wild-type protein at pH 2.5 were next added to each reaction to determine the apparent elongation rate of each variant from a common template (Materials and Methods). Under these conditions, highly reproducible kinetics ensue, allowing accurate determination of the apparent elongation rates^19^ and a more detailed comparison of the influence of different amino acid substitutions on the aggregation kinetics. All variants were able to extend seeds made from wild-type β_2_m, consistent with each variant assembling into a common fibrillar architecture (Fig. 4b). These experiments revealed that variants located predominantly in one region of the sequence (residues ∼ 60–70) reduce the apparent elongation rate with respect to the wild-type protein (Fig. 3c and d and Supplementary Table 1), highlighting the key role of this region in determining both the length of the lag time and the apparent rate of elongation. Three further variants, V27A, F56A and P72G, also affected the apparent elongation rate, suggesting that these residues are also important in the elongation process.

The combined results from both sets of experimental data demonstrate the remarkable and unexpected result that only one region of contiguous amino acids (residues ∼ 60–70) within intact denatured β_2_m plays a pivotal role in determining the rate of fibril nucleation and elongation at low pH. This finding is in marked contrast with predictions of intrinsically amyloidogenic regions based on the physicochemical properties of the amino acid sequence^25–27^ and experimental analyses using peptide fragments (Fig. 2b). Akin to a protein folding reaction,^37^ therefore, the kinetics of fibril formation seem to be regulated by a few key residues, with the rest of the sequence providing a scaffold for encouraging profitable interactions.

### Non-random structure within denatured β_2_m modulates its aggregation propensity

**Fig. 5:**
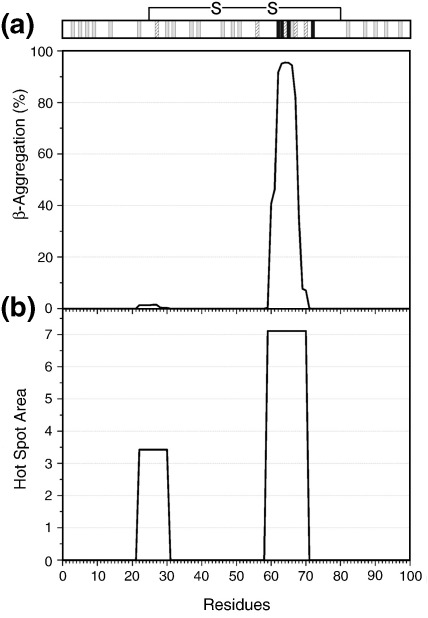
The aggregation propensity of the primary sequence of β_2_m predicted by the algorithms (a) TANGO^26^ and (b) Aggrescan.^27^ A schematic illustration of the effects of amino acid substitution on the rates of fibril elongation determined here is shown above. The effects are classified into three groups dependent on the change in apparent elongation rate; > 2-fold change, grey; intermediate, 2- to 4-fold change, hashed; slow, > 4-fold change, black.

**Fig. 6:**
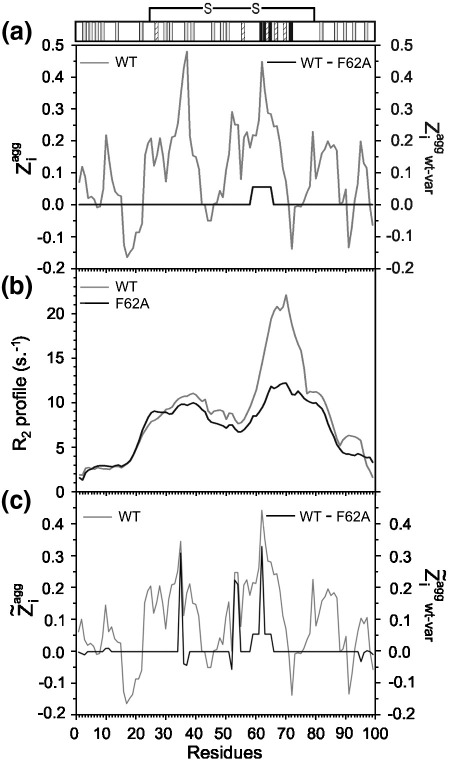
Prediction of the aggregation properties of β_2_m and an example variant, F62A. (a) Intrinsic propensity for aggregation of wild-type β_2_m (grey, left axis) as predicted by the Zyggregator algorithm modified to acidic conditions.^25^ The difference in aggregation propensity between wild-type β_2_m and its variant F62A (black, right axis). F62A is predicted by $Z_{\text{i}}^{\text{agg}}$ to have only a small local effect on the intrinsic aggregation propensity of β_2_m. A schematic illustration of the observed effects of amino acid substitution on the apparent rate of fibril elongation taken from Fig. 3d is shown above. The effects are classified as in Fig. 3, with rates not significantly altered compared with wild-type β_2_m (grey), 2- to 4-fold change (hashed) and > 4-fold change (black). (b) Comparison of the smoothed *R*_2_ relaxation rates for wild-type β_2_m and F62A β_2_m. Plots of *R*_2_ rates on a per-residue basis for all variants are shown in Fig. 8. (c) Comparison of the aggregation propensities of wild-type and the difference in aggregation propensities between wild-type and F62A β_2_m obtained by modification of the Zyggregator algorithm by the measured *R*_2_ values (${\tilde{Z}}_{\text{i}}^{\text{agg}}$).

The ability to form fibrils is thought to be a common property of most polypeptide chains, as long as suitable environmental conditions are found.^3^ Conditions that result in the exposure of the polypeptide chain to solvent thus reveal its inherent aggregation propensity, a property that is dependent on the intrinsic physicochemical properties of the amino acids in the sequence.^3^ These properties have been used as a basis for the formulation of phenomenological models capable of predicting changes in aggregation propensity upon mutation, as well as the absolute rates of aggregation of peptides and unstructured polypeptide chains.^26,27,35,38^ Predictions of the aggregation-prone regions of β_2_m using TANGO, Aggrescan and Zyggregator^25–27^ identified the regions ∼ 20–40 and ∼ 50–70 as having high intrinsic aggregation propensities (Figs. 5 and 6a), albeit with differences in the magnitude of the aggregation propensity for each region. The prediction of residues ∼ 50–70 as the most aggregation-prone region is in agreement with the experimental data presented here (Fig. 3b and d and Supplementary Table 1) and with those previously presented.^19,22^ Interestingly, of the six residues mutated in region 20–40 (F22A, V27A, F30A, P32G, V37A and L39A), a sequence that is predicted to have a high intrinsic aggregation propensity by two of the algorithms (Aggrescan and Zyggregator) (Figs. 5 and 6a) and supported using peptide studies^23,29^ (Fig. 2b), only one mutation (V27A) caused a change (∼ 2-fold) in the apparent elongation rate, and none significantly affected the lag time (Fig. 3b and d). It is therefore important to understand why the intrinsic aggregation propensity is not by itself a good predictor of the tendency to aggregate for this region of β_2_m. In the case of fully folded proteins, we already demonstrated that regions that have a high intrinsic aggregation propensity might not play an important role in the aggregation process because they have also a high propensity to form intramolecular interactions in the native structure and, hence, be protected, at least in part, from giving rise to aggregation.^11^ We therefore investigated here whether intramolecular interactions are also able to alter the intrinsic aggregation propensities under non-native conditions. In the case of β_2_m, the region comprising residues ∼ 20–40, which has an intrinsic aggregation propensity predicted by Zyggregator similar to that of residues ∼ 50–70 (Fig. 6a), not only contains significant non-native structure in the acid-denatured state of β_2_m^19,21^ but also contains Cys25, a residue that forms a disulfide bond with Cys80 that is essential both for the formation of a stable native structure and the generation of amyloid fibrils from full-length β_2_m.^39,40^ Given that fragment 20–41 (known as the K3 peptide) has been shown to form long, straight amyloid fibrils under acidic conditions either in isolation^29^ or when disulfide bonded to its complementary 76–91 fragment,^23^ the data suggest that this region is protected from aggregation by residual structure and/or the presence of a disulfide bond^41^ in the acid-denatured state of the full-length protein.

**Fig. 7:**
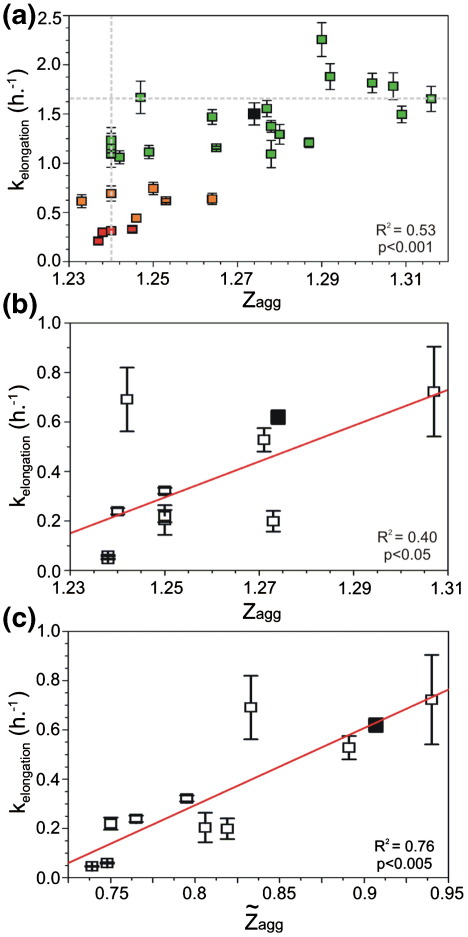
Correlation of the predicted and observed rates of aggregation for β_2_m and its variants. (a) The observed elongation rates of wild-type β_2_m and its 30 variants (measured at 37 °C) compared with their predicted *Z*_agg_ scores calculated at low pH. The data are coloured according to Fig. 3d. (b) Comparison of the observed elongation rates of wild-type β_2_m and 10 variants (measured at 25 °C) with the predicted *Z*_agg_ scores at low pH; the red line indicates the correlation. (c) ${\tilde{Z}}_{\text{agg}}$ scores (including the modulation of the *Z*_agg_ scores through the use of *R*_2_ relaxation rates) of (b), the outlier where *k*_elong_ = 0.69 corresponds to F30A. The red line indicates the correlation. In all plots, wild-type β_2_m is represented as a black square.

To further explore the hypothesis that non-random structure in the acid-unfolded state of β_2_m influences fibril formation, we compared the apparent rate of fibril elongation determined from seeded growth experiments with predictions derived using the Zyggregator algorithm modified to account for the low pH conditions^25^ (Figs. 6a and 7a). Using this algorithm, the agreement between the predicted and observed aggregation rates was significant (*p* < 0.001) but not high (*R*^2^ = 0.53) (Fig. 7a). For example, five variants (V27A, V37A, Y63A, Y66A and V82A) are predicted to have an identical $Z_{\text{i}}^{\text{agg}}$ score (1.24), yet they vary by 4-fold in their observed elongation rates (Fig. 7a, vertical grey line). Additionally, variants F22A and I46A have similar experimental rates of elongation (∼ 1.68 h^− 1^), but are predicted to have $Z_{\text{i}}^{\text{agg}}$scores that differ substantially (1.25 and 1.32, respectively) (Fig. 7a, horizontal grey line). These data indicate that the prediction of intrinsic propensity for aggregation from the inherent properties of the constituent amino acids should be coupled to an assessment of the tendency to form intramolecular interactions to accurately define the aggregation properties of β_2_m at pH 2.5.

**Fig. 8:**
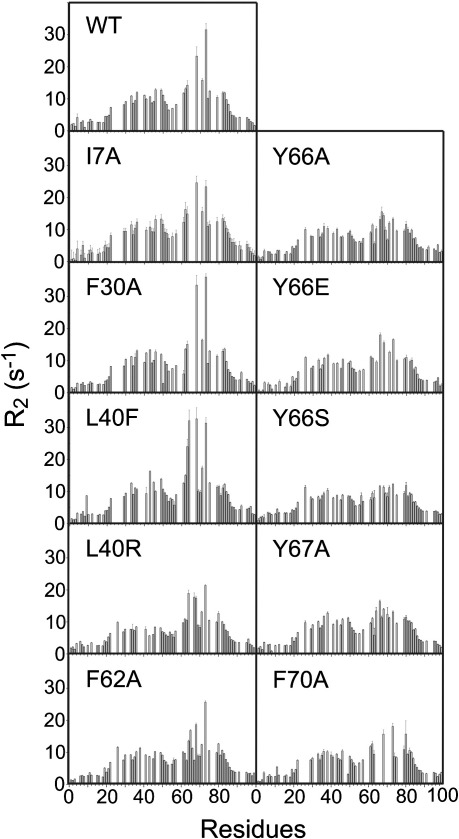
Residue-specific *R*_2_ rates of wild-type β_2_m and 10 variants at pH 2.5 and 25 °C. *R*_2_ rates were measured at 500 MHz in water as described in Materials and Methods.

As it has been observed by NMR measurements that acid-unfolded β_2_m contains significant non-random hydrophobic interactions,^21^ we hypothesised that these intramolecular interactions, in addition to the presence of the disulfide bond, could modulate the aggregation potential of the denatured chain. To account for this, we further generalised the Zyggregator algorithm to include the presence of intramolecular interactions, indicated by the magnitude of the *R*_2_ values measured on a per-residue basis using NMR (Figs. 6b and 8) (Materials and Methods). The use of the *R*_2_ relaxation data to account for the tendency to form intramolecular interactions in the low pH state of β_2_m is analogous to the use of protection factors from hydrogen exchange to rationalise the effects of the protection provided by the burial of the amyloidogenic regions in the native state described previously.^25^ Modification of $Z_{\text{i}}^{\text{agg}}$ by the *R*_2_ value (termed ${\tilde{Z}}_{\text{i}}^{\text{agg}}$) also takes into account structural restraints dictated by the disulfide bond and allows the effect of specific amino acid substitutions on the intrinsic aggregation potential to be modulated by the conformational properties of the precursor state and its sensitivity to amino acid substitutions (Fig. 6c). For the analysis, we utilised a test set consisting of wild-type β_2_m and 10 variants (I7A, F30A, L40F, L40R, F62A, Y66A, Y66E, Y66S, Y67A and F70A) (Supplementary Table 2) for which both the *R*_2_ values and the apparent elongation rates determined using seeded reactions (at 25 °C) were available (Fig. 8).^19^ Excluding the contribution of the NMR *R*_2_ data (Fig. 7b), the correlation between the experimentally determined apparent elongation rates and those predicted using the low-pH, modified Zyggregator algorithm was not high (*R*^2^ = 0.40) (Fig. 7b), indicating that the role of intramolecular interactions should be accounted for in order to perform accurate predictions. The incorporation of the *R*_2_ measurements for each variant (Figs. 6c and 8) into the Zyggregator algorithm (see Materials and Methods) results in a significant improvement in the correlation with the corresponding experimental values (*p* < 0.005, *R*^2^ = 0.76) (Fig. 7c), although some of the rates in the ∼ 20–40 region are still overestimated (particularly for F30A), indicating that the *R*_2_ measurements used here provide only an approximate estimation of the role of intramolecular interactions in modulating the intrinsic aggregation propensities. In the case of native states, we were able to show that the tendency to form intramolecular interactions could be predicted directly from the sequence, without the need of measuring protection factors from hydrogen exchange.^11^ It could also be possible to estimate these tendencies in non-native states, thus avoiding the measurements of *R*_2_ relaxation rates to perform the predictions of the overall aggregation propensities for different protein sequences. The results demonstrate that the residual structure in acid-denatured β_2_m is important in modulating the inherent aggregation propensity endowed by its amino acid sequence and that the ability of this structure to enhance or retard the observed aggregation rate is dependent upon the location of intramolecular interactions in the polypeptide chain. They also show how the inclusion of information describing these structural characteristics permits accurate prediction of the effect of even subtle mutations on the rate of fibril formation.

## Discussion

The results presented show that only one of the intrinsically aggregation-prone regions of β_2_m predicted using algorithms or peptide studies defines the aggregation potential of the complete polypeptide chain under the denaturing conditions that we have examined. This finding indicates that intramolecular and intermolecular interactions compete against each other to determine the overall aggregation process even in denatured proteins, so that the intrinsic propensities for aggregation and for forming soluble monomeric species modulate each other. Indeed, previous studies have implicated long-range intramolecular contacts involving the NAC region of α-synuclein in defining its aggregation properties,^42,43^ whilst the presence of one particular hydrophobic cluster in denatured lysozyme at pH 2 has been shown to play a major role in preventing fibril formation.^44^ As we have demonstrated here, incorporation of the competing influences on collapse, folding and aggregation (Fig. 1) into prediction algorithms enables regions critical for aggregation to be identified in the context of full-length polypeptide sequences and the effects of subtle mutations to be quantified. Thus, whilst the intrinsic aggregation propensity can provide information as to which regions of the sequence have a potential to aggregate, any consideration of fibril formation rates must be careful to take the structural properties of monomeric and oligomeric species into account. Taken together, these insights provide a deeper understanding of the fundamental forces that control the competition between folding, misfolding and aggregation. In addition, they should facilitate the development of rational design strategies to identify reagents capable of inhibiting aggregation by targeting regions known to be critical for the aggregation process.

## Materials and Methods

The *Escherichia coli* strain BL21(DE3) pLysS was obtained from Promega. Q-Sepharose and all other reagents were purchased from the Sigma-Aldrich Chemical Company. Spectrapore dialysis membrane (molecular mass cutoff, 3500 Da) was acquired from Spectrum Laboratories, Inc. Superdex 75 was purchased from Amersham Biosciences. Carbenicillin was obtained from Melford Chemicals. Deuterated solvents were purchased from Fluorochem Ltd., and oligonucleotides were from MWG Biotech.

### Generation of variants and protein purification

Single amino acid substitutions and the variant, ΔN6, were generated within the template gene pINK as previously described.^19,45^ ΔC83 was created by replacement of the codons for His84 and His85 with stop codons. Unlabelled and ^15^N-labelled proteins were expressed and purified to ≥ 95% purity as previously described.^19^

### Kinetic analysis of fibril formation

Data were recorded as previously described^19^ with small variations in the methodology. Briefly, stock solutions of protein in ∼ 5 mM HCl were centrifuged at 543,000***g*** for 1 h (20 °C) and only the upper two-thirds of the solution was used for fibril growth experiments. For spontaneous fibril growth reactions, 84 μM β_2_m (*n* = 6 and *n* = 9 for wild-type and variant β_2_m samples, respectively) was incubated in pH 2.5 buffer (25 mM sodium phosphate, 25 mM sodium acetate and 0.02% azide) containing 10 μM ThT with agitation (600 rpm) in the wells of 96-well black-wall plates (Costar) and sealed with clear sealing film (Axygen). Measurements were recorded at 25 or 37 °C, as reported. ThT fluorescence was recorded at 5-min intervals using a FLUOstar Optima reader (BMG) as previously described.^19^ Comparison of simultaneous ThT and intrinsic tryptophan fluorescence measurements showed that ThT did not interfere with fibril formation, and its fluorescence is a reliable probe of fibril formation (data not shown).^20^ ΔC83 formed fibrils after incubation of the same samples at 200 rpm for > 4 weeks at 37 °C.

In the case of seeded reactions at 37 °C, 5% (v/v) seed was added to each reaction and fibril growth was monitored as described above. The seed consisted of wild-type β_2_m fibrils, formed in pH 2.5 buffer, agitated (200 rpm) for 1 week at 37 °C and subsequently fragmented by three freeze–thaw cycles. The seeds (Fig. 4b) were stored at − 80 °C, and the same batch of seeds was used for all reactions. For comparison with NMR measurements, the apparent rates of elongation determined using seeded reactions of the second test set of variants were measured at 25 °C with the addition of 2% (v/v) wild-type seed.^19^

All data were normalised to the final ThT signal (after 72 h growth), and the resulting curves were used to determine the lag times and apparent rates of elongation. The lag time was obtained by fitting a straight line to the slope of the growth phase between approximately 30% and 70% of the maximum amplitude, and the time at which this line intersected the baseline was taken as the lag time (Fig. 3b, inset). The apparent elongation rates (*k*) were determined by fitting a tangent to the initial part of the growth curve (Fig. 3d, inset).

### Electron microscopy

Samples were examined either undiluted or with the use of a 1:10 dilution in 5 mM HCl on freshly ionised formvar- and carbon-coated electron microscopy grids (Agar). The grids were rinsed with 5 mM HCl and stained with 4% (w/v) uranyl acetate. All images were taken using a Philips CM10 electron microscope operating at 80 keV.

### NMR spectroscopy

All NMR experiments were carried out using 5 mg ml^− 1 15^N-labelled β_2_m in 90% (v/v) H_2_O/10% (v/v) ^2^H_2_O at pH 2.5 and 25 °C. Experiments were performed using a Varian Unity Inova spectrometer operating at a proton frequency of 500 MHz. Gradient-enhanced ^1^H–^15^N heteronuclear single quantum coherence spectra were acquired using 128 complex points and 16 scans per increment with spectral widths of 4508 and 1200 Hz in the ^1^H and ^15^N dimensions, respectively. Watergate solvent suppression was used, and all NMR data were processed using NMRPipe.^46^ The data were apodised using a cosine bell function, followed by zero filling and Fourier transform. The 2D spectra were analysed in NMRView.^47^ Backbone ^15^N transverse *R*_2_ relaxation measurements were carried out using a series of 11 experiments with mixing times ranging from 16.32 to 456.96 ms, as previously described.^19,48^ Duplicate points and the spectral noise levels were used to obtain an estimate of the error.

### Calculation of the aggregation propensities

The aggregation propensities at acidic pH were calculated using *Z*_agg_ scores as described by Tartaglia *et al*.,^11^ but using pH-dependent propensity scales.^25^ In our previous work, the intrinsic aggregation propensities due to the presence of residual structure were modulated through the use of hydrogen exchange profiles.^11^

$${\bar{Z}}_{\text{i}}^{\text{agg}}=Z_{\text{i}}^{\text{agg}}(1-{ɛ\text{HX}}_{\text{i}})$$

where ε = 1/15 and HX_i_ is the protection factor of residue i.^11^

In analogy with this formula, we used here the *R*_2_ relaxation rates for modulating the aggregation propensities. The *R*_2_ data were smoothed using a seven-residue window and labelled as ρ_i_. The $Z_{\text{i}}^{\text{agg}}$ profiles were modified by taking into account the *R*_2_ data using the following phenomenological formula:

(1) $${\tilde{Z}}_{\text{i}}^{\text{agg}}=\{\begin{array}{c} \left(Z_{\text{i}}^{\text{agg}}-\alpha \rho_{\text{i}}\right)*\left(1+\alpha \frac{\rho_{\text{i}}}{\rho_{\text{max}}}\right)\;\text{if}\;Z_{\text{i}}^{\text{agg}}>0\\ Z_{\text{i}}^{\text{agg}}\;\text{otherwise} \end{array}$$

where the constant α = 1.32 and ρ_max_ = 30 represents the maximum of ρ_i_.

The analytical form of the equation captures the fact that regions such as 20–40 and 50–70 are involved in both aggregation and folding. As indicated in the term $Z_{\text{i}}^{\text{agg}}-{\alpha \rho }_{\text{i}}$, the aggregation propensity decreases as a consequence of the propensity to form native contacts, whilst the multiplicative term $\left(\text{1}+\alpha \frac{\rho_{\text{i}}}{\rho_{\max}}\right)$ introduces the effect that an intrinsic propensity to form intramolecular contacts comes at the cost of an increased propensity to form intermolecular contacts. Here, Eq. (1) represents the expansion of our previous formula^11^ that allows the calculation of the aggregation propensity using the structural information based on the conformation properties of the precursor state.

The global score is calculated as follows:^25^

$${\tilde{Z}}_{\text{agg}}=\frac{\sum_{i=1}^{\text{length}}{\tilde{Z}}_{\text{i}}^{\text{agg}}*\theta ({\tilde{Z}}_{\text{i}}^{\text{agg}})}{{\left(\sum_{i=1}^{\text{length}}\theta ({\tilde{Z}}_{\text{i}}^{\text{agg}})\right)}^{\gamma }}L^{\beta }$$

where β = 0.44 and γ = 0.75 and *L* is the length of the amino acid sequence.

The function $\theta ({\tilde{Z}}_{\text{i}}^{\text{agg}})=1$ if ${\tilde{Z}}_{\text{i}}^{\text{agg}}>0$ and $\theta ({\tilde{Z}}_{\text{i}}^{\text{agg}})=0$ otherwise.
