# Supplementary material for: Competition between Intramolecular and Intermolecular Interactions in an Amyloid-Forming Protein
Source: J Mol Biol. 2009 Jun 19;389(4):776–86. doi: 10.1016/j.jmb.2009.04.042 (PMC2722902; doi:10.1016/j.jmb.2009.04.042)
Supplement: Supplementary material [file mmc1.doc]

**Competition Between Intra-Molecular and Inter-Molecular Interactions in an Amyloid Forming Protein**

Katy E. Routledge^1^, Gian Gaetano Tartaglia^2^, Geoffrey W. Platt^1^, Michele Vendruscolo^2^,* and Sheena E. Radford^1^,*

**Supplementary Information**

**Supplementary Table 1.** **Measured parameters of fibril formation by β_2_m variants at pH 2.5, 37 °C. Lag_variant_ /Lag_wild-type_ values and k_wild-type_/k_variant_ values of the non-seeded growth and seeded growth, respectively, each normalised to the values for the wild-type protein. The conditions used are given in the main text Methods section. One standard deviation from the mean is given in brackets.**

| **Variant** | **Lag_variant_ /Lag_wild-type_ (± S.D.)** | **k_wild-type_/k_variant_ (± S.D.)** |
| --- | --- | --- |
| **WT** | 1.00 (0.14) | 1.00 (0.11) |
| **ΔN6** | 0.83 (0.11) | 0.92 (0.09) |
| **R3A** | 0.82 (0.44) | 0.80 (0.07) |
| **P5G** | 1.10 (0.13) | 1.09 (0.09) |
| **I7A** | 1.06 (0.21) | 0.84 (0.07) |
| **V9A** | 0.63 (0.30) | 0.96 (0.09) |
| **P14G** | 1.45 (0.50) | 1.24 (0.11) |
| **F22A** | 1.11 (0.18) | 0.89 (0.07) |
| **V27A** | 0.77 (0.15) | 2.17 (0.29) |
| **F30A** | 1.58 (0.23) | 1.42 (0.11) |
| **P32G** | 1.23 (0.35) | 1.00 (0.09) |
| **V37A** | 1.67 (0.45) | 1.28 (0.12) |
| **L39A** | 0.50 (0.08) | 1.02 (0.09) |
| **I46A** | 0.67 (0.11) | 0.90 (0.08) |
| **V49A** | 1.13 (0.16) | 0.83 (0.08) |
| **H51A** | 3.51 (0.64) | 1.35 (0.13) |
| **F56A** | 1.95 (0.18) | 3.39 (0.40) |
| **F62A** | 5.64 (1.31) | 5.02 (0.83) |
| **Y63A** | 2.41 (0.33) | 4.77 (0.74) |
| **L64A** | 1.22 (0.36) | 2.44 (0.32) |
| **L65A** | 6.69 (1.04) | 7.20 (1.26) |
| **Y66A** | 1.70 (0.43) | 1.37 (0.20) |
| **Y67A** | 1.95 (0.41) | 2.02 (0.23) |
| **F70A** | 2.00 (0.22) | 2.36 (0.28) |
| **P72G** | 1.76 (0.19) | 4.56 (0.52) |
| **V82A** | 0.22 (0.03) | 1.22 (0.12) |
| **L87A** | 1.52 (0.37) | 0.71 (0.06) |
| **P90G** | 1.31 (0.38) | 1.16 (0.13) |
| **V93A** | 1.67 (0.45) | 1.29 (0.11) |
| **R97A** | 1.85 (0.29) | 1.30 (0.12) |
| **ΔC83** | N.D. | 2.42 (0.20) |

**Supplementary Table 2. Rate of fibril elongation of β_2_m variants at pH 2.5, 25 °C.** The apparent elongation rates (s^-1^) of wild-type β_2_m and 10 variants determined by seeded growth at pH 2.5, 25 °C using seeds formed from wild-type protein (see Methods, main text). One standard deviation from the mean is given in brackets.

| **Variant** | **k_elongation_ (± S.D.)** |
| --- | --- |
| **WT** | 0.62 (0.03) |
| **I7A** | 0.72 (0.18) |
| **F30A** | 0.69 (0.13) |
| **L40F** | 0.53 (0.05) |
| **L40R** | 0.20 (0.04) |
| **F62A** | 0.06 (0.003) |
| **Y66A** | 0.24 (0.01) |
| **Y66S** | 0.32 (0.01) |
| **Y66E** | 0.20 (0.06) |
| **Y67A** | 0.22 (0.02) |
| **F70A** | 0.05 (0.002) |
